# First four years of operation of a municipal acute bed unit in rural Norway

**DOI:** 10.1080/02813432.2018.1523993

**Published:** 2018-10-05

**Authors:** Anne Kjær Schmidt, Bård Lilleeng, Valborg Baste, Thomas Mildestvedt, Sabine Ruths

**Affiliations:** a Research Unit for General Practice, Uni Research Health, Bergen, Norway;; b Luster Legekontor, Luster, Norway;; c Uni Research Health, Bergen, Norway;; d Department of Global Public Health and Primary Care, University of Bergen, Bergen, Norway

**Keywords:** Emergencies, intermediate care facilities, primary care, hospitals, community, Norway

## Abstract

**Objective:** To evaluate the use of a small municipality acute bed unit (MAU) in rural Norway resulting from the *Coordination reform* regarding occupancy-rate, patient characteristics and healthcare provided during the first four years of operation. Further, to investigate whether implementation of the new municipal service avoided acute hospital admissions.

**Design:** Observational study.

**Setting:** A two-bed municipal acute bed unit.

**Subjects:** All patients admitted to the unit between 2013 and 2016.

**Main outcome measures:** Demographics, comorbidity, main diagnoses and level of municipal care on admission and discharge, diagnostic and therapeutic initiatives, MAU occupancy rate, and acute hospital admission rate.

**Results:** Altogether, 389 admissions occurred, 215 first-time admissions and 174 readmissions. The mean MAU bed occupancy rate doubled from of 0.26 in 2013 to 0.50 in 2016, while acute hospital admission rates declined. The patients (median age 84.0 years, 48.9% women at first time admission) were most commonly admitted for infections (28.0%), observation (22.1%) or musculoskeletal symptoms (16.2%). Some 52.7% of the patients admitted from home were discharged to a higher care level; musculoskeletal problems as admission diagnosis predicted this (RR =1.43, 95% CI 1.20–1.71, adjusted for age and sex).

**Conclusion:** Admission rates to MAU increased during the first years of operation. In the same period, there was a reduction in acute hospital admissions. Patient selection was largely in accordance with national and local criteria, including observational stays. Half the patients admitted from home were discharged to nursing home, suggesting that the unit was used as pathway to a higher municipal care level.

**Key Points**
Evaluation of the first four years of operation of a municipality acute bed unit (MAU) in rural Norway revealed:
• Admission rates to MAU increased, timely coinciding with decreased acute admission rates to hospital medical wards.
• Most patients were old and had complex health problems.
• Only half the patients were discharged back home; musculoskeletal symptoms were associated with discharge to a higher care level.

## Introduction

In many countries, demographic changes including more patients living longer with chronic health problems put an increasing burden on health services, in particular hospital services [1]. The implementation of the Norwegian *Coordination Reform* (2012) aims at developing and strengthening the municipal health services, thus reducing acute hospital admissions [2]. As part of the reform, municipality acute bed units (MAUs) were established for selected patients who otherwise would have been admitted to hospital [3]. The new service is intended for short-term stays of patients diagnosed with acute conditions manageable by primary care methods, or chronic conditions in need of re-evaluation of treatment [3]. MAUs were established in many municipalities from 2012 and mandated by the Government from 2016. The units are usually co-located with local nursing home wards or out-of-hours (OOH) emergency services [4]. Previous research suggests that MAUs were initially underused [5] but contributed to reducing acute medical admissions [6]. Patients admitted to MAUs are slightly more satisfied as compared to patients admitted to hospitals, but evidence is sparse with regard to clinical outcomes [7].

To the best of our knowledge, no studies have evaluated time trends in the use of MAUs. This information is relevant for dimensioning and tailoring this new service according to patients’ needs. Consequently, we evaluated trends in using a small MAU in rural Norway with regard to occupancy-rate, patient characteristics and healthcare provided during the first four years of operation. Further, we investigated whether the implementation of this municipal service was associated with reductions in acute hospital admissions.

## Material and methods

### Study population

This observational study was conducted in a single municipality in north-western Norway with approximately 5200 inhabitants. The municipality established a two-bed MAU in 2012, equaling a bed rate of 0.38/1000 inhabitants, compared to the national norm of 0.13/1000. Although the formal opening of the MAU took place in December 2012, the unit was put into use already earlier the same year. The unit was centrally located in the municipality, with patient transfer time to hospital of about two hours by car and 20 minutes by helicopter, weather permitting.

The MAU was co-located with the municipal nursing home and OOH emergency service. In addition to the MAU, the short-term nursing home ward comprised 12 beds assigned to rehabilitation after discharge from surgical and medical hospital departments, and patients in need of palliative care. Patient could be admitted directly to the different types of short-term beds. The only general practitioner (GP) office in the municipality was located close to the MAU. Medical care was provided by one GP employed at the MAU every weekday and by alternating GPs on call after hours and in the weekends. All admitted patients were examined by doctor and physiotherapist within the first 24 hours, and twice a week discussed and reviewed by a multidisciplinary team consisting of doctor, nurse, physiotherapist and occupational therapist. The unit had access to equipment for primary medical care examinations, laboratory services (e.g. hemoglobin, WBC differential, CRP, glucose and urine examinations), ECG and bladder scan. Other blood tests had to be examined at the local hospital laboratory and test results would be received electronically the next day or later. Patients had to be transferred to the local hospital when in need of X-ray.

To avoid patients in need of hospital care being admitted to MAU, local inclusion criteria were set up (Supplementary table). It was up to the admitting doctor to decide whether the patient fulfilled the MAU criteria or should be admitted to hospital instead. All admission decisions would be reviewed at the GPs’ staff meeting held the following morning on weekdays.

In June 2017 an information letter was sent to all 217 patients admitted to MAU during the study period, from January 2013 to December 2016. The study population comprised all eligible patients, except two patients who declined participation.

### Data collection

For all stays in the MAU 2013–2016, routinely registered information was extracted from patients’ electronic medical record and transferred to a data sheet. We included administrative data (date of admission and discharge, municipal care level on admission and discharge, referring doctor), demographics (age, gender), and clinical data (diagnostic group on admission and discharge, comorbidities, diagnostic and therapeutic initiatives, modified early warning score (MEWS), and activities of daily living (ADL) score on admission and discharge). To keep track of readmissions, the head nurse in charge of the MAU replaced patients’ ID-number with a record number on the data sheet, the key to which remained undisclosed to the research group. Occupancy rate was defined as the total use of beds (days) divided by total available bed days annually.

From Helse F⊘rde Hospital Trust [8] we obtained numbers of acute medical admissions to the local hospital that the patients admitted to MAU otherwise would have been admitted to. In addition equivalent numbers for the county and Norway was obtained. These numbers are presented per 1000 inhabitants, for each year 2010–2016.

### Statistical analyses

Due to the exploratory nature of this study, no power analysis was performed. Descriptive statistics are presented as median, range, mean, standard deviation (SD) and percentages. Differences in mean values were tested with analyses of variance (ANOVA). Differences between years of admission and categorical variables were tested with chi-square tests. To investigate possible association between diagnostic groups (infections, observation and musculoskeletal) and higher care level on discharge, log-binomial regression was performed to estimate relative risks (RR) with 95% confidence interval (CI). All analyses were performed with IBM SPSS Statistics 24.

## Results

Altogether 389 admissions occurred during the study period, 215 first-time admissions; 91 of these patients were admitted more than once and represent 174 readmissions. Annual admission numbers increased from 81 in 2013 to 115 in 2016 and mean annual bed occupancy rate increased from of 0.26 to 0.50. Length of stay varied between 0.5 and seven days (median 3), and mean length of stay increased over the years (*p* = 0.005). Most patients were admitted by doctors on call OOH (84.6%), 11.3% by GPs and some 4.1% from hospital or home nursing service, Table 1. The latter group typically comprised patients in assisted living arrangements adjacent to the MAU admitted during the night and awaiting examination by the doctor the next day. Patients from hospital were admitted with minor injuries but no fractures after falling, for pain-relief and mobilisation.

**Table 1.** Admissions to municipal emergency beds (*n* = 389).

			Length of stay	
Admission year	*n*	%	Mean	SD
2013	81	20.8	2.4	0.9
2014	85	21.9	2.7	1.2
2015	108	27.8	3.0	1.3
2016	115	29.6	2.8	1.3
Admission time				
Work day, day 8:00 AM – 3.30 PM	144	37.0		
Work day, evening 3.30 PM – 11:00 PM	137	35.2		
Work day, night 11:00 PM – 8:00 AM	35	9.0		
Weekend/Public holiday	73	18.8		
Referring doctor				
Doctor at out-of-hours service	329	84.6		
General practitioner	44	11.3		
Others (hospital doctor, home nurse)	16	4.1		

At first-time admission, patients’ median age was 84.0 years (range 20–102), 48.9% were women; they had on average 1.7 preexisting comorbidities (range 0–5), cardiovascular disease being most prevalent (Table 2), none of these variables changed over the four years. Regarding all admissions, patients were most commonly admitted for infections (total 28.0%; pneumonia 13.1% and urinary tract infection 6.2%) but decreased from 40.7% in 2013 to 23.5% in 2016 (*p* = 0.01). In 22.1% of the cases, the patient was admitted for observation and 16.2% for musculoskeletal symptoms. Compared to patients younger than 75 years, those aged 75+ were more commonly admitted for musculoskeletal symptoms (*p* = 0.04) and less commonly for observation (*p* = 0.04); they had longer stays (*p* < 0.001), higher scores on MEWS (*p* = 0.02), and ADL on admission (*p* < 0.001) and discharge (*p* < 0.001); they had more comorbidities (*p* = 0.01) and more commonly cardiovascular disease (*p* < 0.001) or dementia (*p* = 0.02).

**Table 2.** Patient characteristics of first time admission by age group, and all admissions to a municipal acute bed unit in the period 2013–16.

	First time admission (*n* = 215)					
	Age <74 y		Age ≥75 y		All admissions (*n* = 389)	
	*n*	%	*n*	%	*N*	%
Sex						
Male	26	51.1	94	57.3	194	50.1
Female	25	49.0	70	42.7	195	49.9
						
					Mean	SD
Age, years					80.8	13.3
						
Diagnostic group on admission	*n*	%	*n*	%	*n*	%
Infection	15	9.4	54	32.7	109	28.0
Observation	18	35.3	35	21.3	86	22.1
Musculoskeletal	<5		34	20.7	63	16.2
Dehydration	<5		14	8.5	36	9.3
Heart failure	<5		9	5.5	30	7.7
Mental disorder	<5		5	3.0	28	7.2
COPD	<5		8	4.9	17	4.4
Constipation	<5		<5		8	2.1
Social	<5		<5		<5	
Diabetes	<5		<5		<5	
Addiction	<5		<5		<5	
						
	Mean	SD	Mean	SD	Mean	SD
Length of stay in MAU	2.0	1.1	2.7	1.1	2.8	1.2
MEWS^a^, maximum score	1.2	1.3	1.7	1.5	1.7	1.6
ADL^b^ score on admission	2.0	0.9	2.4	0.8	2.5	0.8
ADL^b^ score on discharge	1.8	0.8	2.3	0.9	2.3	0.85
Number of pre-existing comorbidities	1.35	1.07	1.76	0.85	1.8	0.9
						
Pre-existing comorbidities	*n*	%	*n*	%	*n*	%
Cardiovascular	22	43.1	134	81.7	292	75.1
Dementia	<5		36	22.0	83	21.3
Diabetes	10	19.6	34	20.7	80	20.6
Mental disorders	11	21.6	20	12.2	70	18.0
COPD	7	13.7	24	14.7	60	15.4
Cancer	8	15.7	24	14.7	59	15.2
Neurological conditions	5	9.8	16	9.8	55	14.1
Addiction	<5		<5		<5	

^a^MEWS: modified early warning score;

^b^ADL: activities of daily living.

Diagnostic tests and procedures were performed in 97.2% of the cases (Table 3); standard blood samples were taken from almost all patients, while urinary samples and ECG were scarce during the first years and increased to about 90% in 2016. Various treatments were provided; most commonly antibiotics (28.8%), medication adjustments (21.9%) and pain management (21.1%), Table 3. During 26.2% of all admissions, and 44% of admissions for observation, the patients received no specific treatment.

**Table 3.** Diagnostic and therapeutic procedures during stay in municipal emergency beds (*n* = 389).

	*n*	%
Diagnostic procedures		
Blood samples (hemoglobin, CRP, leucocytes, glucose)	378	97.2
Urine samples	212	54.6
ECG	179	46.0
Samples transferred to hospital lab	36	9.3
Glucose profile	23	5.9
Troponin	7	1.8
Observation commotion	6	1.5
INR	5	1.3
Bladder volume	<5	
D-dimer	<5	
No diagnostic initiatives	8	2.1
Supplementary diagnostics		
X-ray at hospital	15	3.9
Consultation hospital specialist (by phone)	59	15.2
Treatments and procedures		
Antibiotics total	112	28.8
Antibiotics intravenous	62	15.9
Other medication adjustments	85	21.9
Pain management	82	21.1
Mobilisation	51	13.1
Intravenous fluid	49	12.6
CPAP	28	7.2
Inhalation therapy	24	6.2
Isolation for infection/Shielding	<5	
Oxygen therapy	11	2.8
Bowel emptying	9	2.3
Catheterization	8	2.1
Wound care	7	1.8
No therapeutic initiative	102	26.2

Excluding four tourists, ten patients who died and one admission lacking discharge information, 197 of 374 (52.7%) patients were discharged to a higher care level than before admission (164 to short-term and 4 to long-term nursing home stay, 29 to hospital), 173 (46.3%) to the same care level, (163 home, 10 long-term nursing home stay) while the four patients admitted from hospital to MAU were discharged to a lower care level. Of the 29 hospitalised patients (mostly for infections), 18 were transferred within one day and seven patients after 2–3 days.

Among diagnostic groups at admission only musculoskeletal symptoms were associated with a higher care level on discharge (RR =1.43, 95% CI 1.20–1.71, adjusted for age and sex).

**Figure 1.**
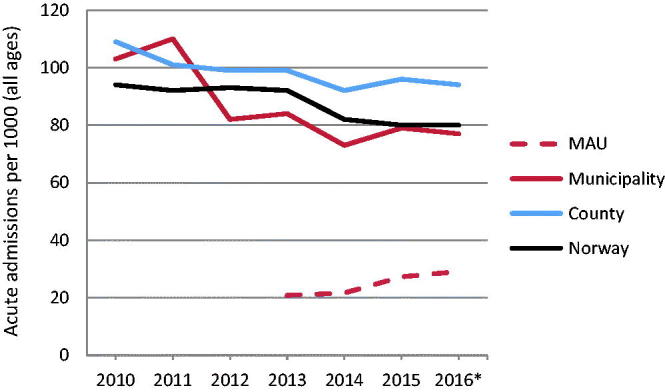
Annual admission rates (per 1000) to medical ward at hospital (acute admissions)** from municipality, county and Norway respectively, and to municipal emergency bed unit (MAU). *Based on the two first tertials; ** Hospital admissions from year 2010 are included to show time trends.

Acute admissions from the municipality to hospital medical wards declined in 2012 and remained stable at a lower level in 2013–2016. A smaller decline was found for hospital admissions from the county and Norway, in 2014 (Figure 1).

## Discussion

### Principal findings

Our study has shown an increasing occupancy rate in the MAU during the first four years of operation. In the same time period, there was a stable lower acute admission rate to medical hospital ward after an initial decline. Most patients were old and had complex health problems. One out of five admissions was for observational purposes and half of these patients received no specific treatment. The length of stay was three days or shorter for half the patients. Of those admitted from home, less than half the patients were discharged back to their home. Admission diagnosis related to the musculoskeletal system was associated with discharge to a higher care level.

### Strengths and weaknesses of the study

This four-year observational study reports complete data from a new health service where there are few studies. We were able to follow all patients from admittance to MAU, to discharge. The main weakness of the study is the restriction to one municipality. Although the sample comprised nearly 400 admissions, some subgroups were too small to examine, such as patients with multiple readmissions. Also, we were unable to validate diagnoses with regard to severity and completeness of information. Numbers on admissions to MAU and hospital respectively were drawn from different sources.

### Findings in relation to other studies

The increasing occupancy rate found during the first four years of operation of the MAU was expected, because it takes some time for referring doctors and the public to grow familiar with a new health service [9]. The occupancy rate in this unit in 2016 (mean 0.49) was higher as compared to the national average in 2015 (mean 0.35, with large variations) [5], but still corresponding to only half the anticipated need. A report by Skinner has pointed at different tradition and organisation of acute care treatment in nursing homes prior to the establishment of MAUs, as explanations of the variation in occupancy rate [10]. Also, in small units that are co-located with nursing homes, the beds can be used flexibly and reported places need not be an absolute limit for capacity.

The approximately 20% decrease in referral rates from the municipality to hospital medical ward (acute admissions) starting in 2012 (Figure 1) supports that the MAU contributes to alleviate the burden on hospital admissions. The unit was put into use at least half a year before the formal opening, associated with a marked decline in hospital admissions in 2012. Our findings align with a population-based study using register-data, demonstrating that the introduction of MAUs in Norway was associated with a small yet significant overall decrease in hospital admissions, especially regarding patients aged 80 years and above [6]. However, we have no control of random variation in acute hospital admissions, or other conditions that may have caused decreased numbers. The concurrence of increasing occupancy rate in the MAU and a stable lower acute admission rates to medical hospital wards did not concur with other administrative changes. Further studies should be accomplished to investigate if this could be due to chance.

The large majority of patients was admitted by doctors on call, in line with previous research [11]. Doctors on call generally have less knowledge of patients and limited information available compared to patients’ regular GP, and this may challenge admission decisions. However, GPs on call in this particular municipality only serve the local community, and they have access to the patients’ medical record. Information on patients’ clinical status and admitting doctors’ considerations prior to admission to MAU were not available, thus doctors’ referral decisions could not be evaluated in this study. But it is known that geographical distance to various acute care services and the GP’s working experience in the local community, are important local factors for the individual doctor’s admission practice [12]. On the other hand, GPs have reported challenges as to whether patients could be considered as “medically clarified” and whether the MAU services were adequate and safe [9,13]. The patient’s and family’s preferences may also have influenced the admission decisions. Patients appreciate MAUs with regards to safety, geographical proximity, treatment facilities and time for care, but perceive the lack of diagnostic resources as a disadvantage [14,15].

Half the patients in our study were admitted for acute conditions related to the respiratory or musculoskeletal system, another 20% for observation; our findings align with national statistics on use of MAUs [5]. Admissions for observation without a specific diagnosis is often appropriate [16] but may put patients at risk of delayed diagnostics and treatment [17,18]. The small rate of patients transferred from MAU to hospital in this study suggests that the risk of delay was lower compared to another small unit [11]. However, we have not examined the quality of treatment provided in this MAU. GPs have reported that they perceive MAUs not merely as an alternative to hospitals, but also as an additional service in cases were the patient is in need of continuous observation and cannot be returned to their home [13]. This applies in particular to very old patients with functional decline. Studies evaluating patient outcomes in MAUs and similar health services, such as community hospitals, compared to general hospital are sparse. A research overview published in 2014 [7] identified only three smaller studies comparing MAUs with hospital care in the UK and Norway [17–19]. Patients admitted to MAUs were slightly more satisfied; however, there is insufficient evidence to determine whether there are differences in patients’ physical function, quality of life or the number of readmissions [7].

Most patients in our study were 80 years and older, and had several co-morbidities. Almost all patients were admitted from home, living independently or assisted (sheltered housing, home care services), but less than half the patients were discharged back to their home. Lappegard found that 70% of the patients were discharged to the same care level as they were admitted from; however, these patients were younger (mean age 73 years), which is important when it comes to functional levels [19]. In our study, musculoskeletal condition as admission diagnosis was the only factor predictive of being discharged to a higher care level. This finding aligns with studies showing that mobility is a critical factor concerning independent living [20]. The patient transfer rate to hospital was lower compared to another MAU [11], but we can only speculate if this is due to more adequate referral decisions, different patient groups or medical treatment available. Transmission was conducted mostly within one day, suggesting a quick identification of needs of specialised health care and low risks of delayed interventions. On the other hand, the large patient transfer rate from MAU to short-time stay in the nursing home may point to needs of further clarification, treatment or rehabilitation. These patients did not have to be moved physically, only administratively, and this may have contributed to the widespread use of this option. We had no access to follow-up data to examine if some of these patients were discharged to their home later. Provided that most patients were very old and had complex health problems, prolonged stays may also indicate that many of them were in need of more comprehensive care, rather than medical treatment [11]. In all cases the unit may have played an important role in multidisciplinary assessment of patients’ functional level and individual needs. Prospective studies are needed to examine other factors predicting patients’ needs of a higher care level after an acute episode.

## Conclusions

Our study revealed increasing admission rates to MAU during the first years of operation. In the same time period there was a reduction in acute hospital admissions. Patient selection was largely in accordance with national and local criteria, including observational stays without therapeutic initiatives. Half the patients admitted from home were discharged to nursing home, suggesting that the unit was used as pathway to a higher municipal care level.
